# Reflection and Foresight on Personal Information Protection and Optimization in Public Health Emergencies in China—From the Perspective of Personal Information Collection during the Period of China’s Dynamic-Zero COVID-19 Prevention and Control Policy

**DOI:** 10.3390/ijerph20021290

**Published:** 2023-01-11

**Authors:** Huimin Wang

**Affiliations:** School of Civil and Commercial Law, Shandong University of Political Science and Law, Jinan 250014, China; wanghuimin@sdupsl.edu.cn

**Keywords:** dynamic zero-COVID-19, China, personal information, optimization, epidemic-related information, information collection, information processing

## Abstract

Public health emergencies threaten the overall public health security of the country. Based on the need to control the ways of infection, the collection and processing of personal information by the government have become an important part of epidemic prevention and control. However, personal information related to the epidemic is highly sensitive, which contains other personal information and even private information in addition to information on personal health. In the early days of China’s response to the public health emergency of COVID-19, a great deal of non-desensitized information was transmitted in an unaccredited manner. With the implementation of epidemic prevention and control measures, the collection and processing of personal information in China have gradually transited from the initial disorder and chaos to the current orderly, legal, and effective situation, continuously optimizing the processing paths of personal information. Serious summary and reflection on the optimization path of China’s epidemic-related information collection and processing methods by looking for a border at which the way and scope of personal information disclosure in future major public health emergencies are compatible with its purpose and role may help to improve the development of China’s personal information protection legal system from a long-term perspective.

## 1. Introduction

After the outbreak of public health emergencies, in order to prevent the spread of the epidemic and ensure the safety of people’s life and health, the most direct and effective measure is to find virus carriers and potential cases in a timely manner and to conduct isolation, observation, and treatment before the final successful development of especially efficient drugs and vaccines. Opposition and complexity exist between the personal interests and social public interests carried by personal information. In particular, personal information in public health emergencies is of great significance for epidemic prevention and control. It is necessary to sort out personal information protection norms, reposition the value and interests of personal information, and balance public interests and personal interests.

The outbreak of the COVID-19 epidemic is seen as “an unprecedented public health and socioeconomic crisis since the end of the Second World War” [1], causing intense anxiety, uncertainty, and controversy [2]. The World Health Organization listed the COVID-19 epidemic as “a public health emergency of international concern” on 30 January 2020, raised its global risk rating from “high” to “very high” on 28 February, and then declared that the epidemic had evolved into “a global pandemic” on 11 March [3]. In China, the National Health Commission of the People’s Republic of China (hereinafter referred to as the “National Health Commission”) made an announcement on 20 January 2020, incorporated COVID-19 into category B infectious diseases stipulated under the *Law of the People’s Republic of China on Prevention and Control of Infectious Diseases* (hereinafter referred to as *Law on Prevention and Control of Infectious Diseases*), and took prevention and control measures for category A infectious diseases [4]. With a wide impact scope, a rapid diffusion rate, and frequent mutation and iteration, this epidemic swept the world in a short time. 

When many countries around the world are faced with the threat of the same virus, the extent of its social and economic impact on each country depends on the understanding of the virus by the government and society in the country and the prevention and control measures taken. Based on differences in political systems, historical traditions, social habits, and cultural orientations, different countries adopted different measures in response to COVID-19 after experiencing the initial panic caused by the rampant spread of the virus.

Ever since the outbreak of COVID-19 at the end of 2019, China has moved quickly from panic to prompt responses, and in accordance with the changes in the epidemic prevention and control situation, orderly realized four different stages from emergency prevention and control, normal prevention and control, precision prevention and control, to comprehensive prevention and control. Among them, the most effective policy for epidemic control is the “dynamic zero-COVID-19” policy. The “dynamic zero-COVID-19” policy aims not to strive for zero infection, but to “exchange space for time”, effectively control the transmission and diffusion scopes of the epidemic, provide timely treatment of existing cases, strengthen vaccination and speed up the research and development of medicines and vaccines within the hard-won time window, and make good preparations for medical resources, isolation beds, effective medicines, material supply, emergency response mechanisms, etc., to gain greater certainty of defeating COVID-19 [5]. Anti-epidemic practice from 2020 to 2022 shows that the dynamic zero-COVID-19 policy has effectively controlled the spread of COVID-19 in China and kept the number of infections and deaths from the disease at a low level, ensuring that the number of infected people and the number of deaths caused by COVID-19 in China are at a low level at a stage when the virus morbidity and mortality are relatively high [6].

Later, in November 2022, after a clearer understanding of its transmission pattern and the characteristics of its clinical treatment, it was determined that the pathogenicity of the variant strain of Omicron had largely weakened. Thus, on 26 December 2022, the National Health Commission of China issued the Notice on the Overall Plan of Managing COVID-19 with Measures against Class B Infectious Diseases” [7], stipulating that measures against Class B infectious diseases will be implemented for COVID-19 infection from 8 January 2023. In accordance with the Law on the Prevention and Control of Infectious Diseases, isolation measures will no longer be implemented for people infected with COVID-19, close contacts will no longer be judged, and high- or low-risk areas will no longer be delimited. The COVID-19-infected persons shall be treated by classification and the medical security policy shall be adjusted in a timely manner. The nucleic acid testing strategy is adjusted to “willing to complete testing”, and the frequency and content of epidemic information release will also be adjusted. In accordance with the Frontier Health and Quarantine Law of China, no measures will be taken to control quarantinable infectious diseases for people and goods entering the country. This means that the focus of epidemic prevention and control in China has shifted from “prevention” to “protection” and “treatment” [8], and the goal of prevention and control should be focused on “protecting health and preventing severe diseases” [9]. Now that the viral morbidity and mortality are deemed under control, China no longer pursues “zero clearing” of positive cases of COVID-19 [10]. 

In the face of such a huge global public health event, no national policy can be perfect [11]. The formulation and implementation of policies at each stage should balance interests under the current situation [12]. Although China’s dynamic zero-COVID-19 has temporarily come to an end, it has played a very important role in the early and middle stages of the COVID-19 epidemic. During the period from December 2019 to December 2022, it effectively prevented the large-scale spread of COVID-19 in China [13], which is a remarkable achievement benefiting from the strict implementation of government policies and dependent on the high degree of cooperation among the public with various policies [14,15].

The key to the success of the dynamic zero-down policy in China lies in the timely grasp and efficient processing of epidemic-related information. The essential requirement is the efficient collection and transmission of personal information. The efficiency of epidemic control has been greatly improved by tracing the source of viruses, tracking-related cases, pre-judging the situation of the epidemic, etc., after the appearance of epidemic-related cases, collecting and processing the geographical location, whereabouts, health status, and other information of relevant individuals, and disseminating relevant information to the masses with the right to know as needed. In the process of regular epidemic prevention, the verification and recording of personal epidemic-related vaccination information, nucleic acid testing information, and whereabouts information also constitute the premise and basis for consolidating the achievements of dynamic zero-COVID-19. Nevertheless, personal epidemic-related information was illegally disclosed and infringed in the name of “epidemic prevention and control” and without due process. Meanwhile, attention needs to be paid to how to deal with a large amount of information collected by some non-epidemic departments during regular epidemic prevention [16].

With the end of dynamic zero-COVID-19, China’s epidemic prevention and control no longer place the collection of epidemic-related personal information in an important position. However, the handling of similar public health emergencies now and in the future can expect the support of big data and information technology. Therefore, it is still necessary for us to seriously summarize and rationally reflect on the epidemic prevention process in the past three years of COVID-19 prevention, and think about how to better collect personal information in public health emergencies and other similar emergencies involving a wide range of groups, so as to systematically improve the protection of personal information in China. It is necessary to face the multiple risks in the field of expanded public health security and personal information protection, as well as the shortcomings in protecting personal information security in practice, summarize and reflect in a timely manner on the measures we have taken in the face of new situations and the experiences and lessons we have learned, and provide useful suggestions for the Chinese government to strengthen personal information protection in future society’s data governance, so as to help improve society’s data governance capability.

## 2. Significance of Collecting and Processing Personal Information in COVID-19 Prevention and Control

Breaking out suddenly, the COVID-19 epidemic is characterized by strong infectivity, rapid diffusion rate, high risk, etc. After the human-to-human transmission nature of the COVID-19 virus was confirmed, the effective control of the COVID-19 epidemic was deemed to be dependent on the timely cutting-off of interpersonal transmission chains and the rapid quarantine and lockdown of infection sources.

### 2.1. Types of Personal Information Involved in COVID-19 Prevention and Control

In June 2020, China published a white paper on *China’s Fight against the Epidemic*, according to the content of which the most direct and effective control measures are to isolate patients in a timely manner, locate potential close contacts and suspected cases in a timely manner, accurately release relevant information, and their track activity in order to cut off transmission routes. In epidemiological treatment, this procedure is called “epidemiological investigation” and requires obtaining the relevant information of people involved in the epidemic, including basic personal identity, social relationships, movement trajectory, and health information to perform investigations from the domains of personal, time, and space [17]. Compared with several previous influential public health events, COVID-19 prevention and control mostly feature the application of big data tools and artificial intelligence in the collection and processing of personal information. Furthermore, this is not a unique operation of the Chinese government. The COVID-19 pandemic led governments around the world to resort to tracking technology and other data-driven tools in order to monitor and curb the spread of the virus [16,18].

*Personal Information Protection Law of the People’s Republic of China* stipulates that personal information refers to all kinds of information related to identified or identifiable natural persons recorded electronically or in other ways, excluding anonymized information (Article 4 of *Personal Information Protection Law of the People’s Republic of China*: Personal information refers to all kinds of information related to identified or identifiable natural persons recorded electronically or in other ways, excluding anonymized information, whose handling includes collection, storage, use, processing, transmission, provision, disclosure, deletion, etc.). Among all sorts of information involved in the epidemiological investigation of the COVID-19 epidemic, basic personal identity information is basic information used to identify individuals, including name, age, contact and biometric information, home address, family members, etc. This class of information contributes to distinguishing one person from another and identifying individuals, mainly concerning information such as the identity of individuals in a group. Personal activity information refers to the relevant information of individuals involved in public or private activities such as asocial activities and communications, mainly including the residence information and activity trajectories of citizens and other people and objects they come into contact with, such as whereabouts routes, residence trajectories, and traffic information. In the epidemiological investigation, personal activity information can be combined with external information to determine when, where, and with whom a person has had close contact to judge the scope of people with potential risk. Personal medical information refers to the medical observation and diagnosis data of individuals, including medical information and records, drug prescriptions, examination results, illness state, medical history, and therapeutic effect. Typically collected by medical institutions receiving and treating patients, such information can be used to determine the type of viruses and the risk of infection, and assess the criticality of public health emergencies and other indicators [19].

### 2.2. Role of Personal Information Collection and Processing in COVID-19 Prevention and Control

In contemporary society, the continuous expansion of economic and cultural interaction and other activities between people, the increasing mobility of people, and the strengthening of trans-regional population mobility invisibly provide convenient conditions for the spread of epidemic diseases. Epidemiological investigations and tracking of cases’ whereabouts are key to epidemic prevention and control. Thus, initiatives supported by information technology and artificial intelligence have been developed by governments and private companies around the world to enable the tracking of the public’s symptoms, contacts, and movements [20,21]. Considering the threats of COVID-19, initiatives designed to support infection surveillance and monitoring are essential and necessary [22,23]. The most important part of this process is the collection and processing of personal epidemic-related information. Data collected during epidemiological investigation play a decisive role in analyzing the transmission mode of epidemics, judging and determining intergenerational transmission, calculating the incubation period, and studying and judging asymptomatic patients. Timely and effective information collection helps epidemic prevention and control departments to obtain information about people involved in the epidemic, learn about the activity trajectories of people, keep the spread of the virus under control as much as possible, and take measures such as treatment, prevention, quarantine, and lockdown for high-risk and susceptible groups as soon as possible, which is the key to the success of the “dynamic zero-COVID-19” prevention and control policy.

The collection and processing of personal information, especially epidemic-related information, have always been a major means of controlling public health events, especially sudden infectious virus epidemics. Yet, the in-depth application of big data tools and artificial intelligence technology in the prevention and control of the COVID-19 epidemic greatly facilitates the acquisition of personal epidemic-related information and saves a lot of manpower and material resources. For example, the information acquisition and screening of confirmed and suspected cases or people who have had contact or may have had contact with them involve a heavy workload and require a high degree of timeliness during epidemic prevention and control. In the absence of modern information technology, different departments may need to use myriads of manpower and material resources for investigation in their respective fields. These include thoroughly searching the travel information of relevant people such as high-speed train, bus, and air tickets using communication signals, some instant messengers or payment tools, and other information of these people for one-to-one tracking, analysis, and screening, and realizing cross-department and even region information sharing, which is laborious, time-consuming, and struggles to meet the needs of timely and effective containment of the epidemic-related scope in COVID-19 prevention and control, and affects the effect of epidemic prevention and control. With the help of big data tools and artificial intelligence technology, however, a standard mode has been formulated for the acquisition of personal epidemic-related information. Basic personal identity, activity, and medical information can be integrated into corresponding programs, which facilitates the unified acquisition and transmission of information and effectively solves the complexity and lag of the aforementioned cross-department, -region, and -channel information acquisition. Hence, the collection, processing, and sharing of personal information during the COVID-19 pandemic have tremendously contributed to the effective implementation of current COVID-19 prevention and control work.

### 2.3. Conflicts between Personal and Public Interests Arising from the Collection and Processing of Personal Information in COVID-19 Prevention and Control

*Personal Information Protection Law of the People’s Republic of China* provides that the personal information of natural persons is protected by law, whose rights and interests may not be infringed upon by any organization or individual (Article 2 of *Personal Information Protection Law of the People’s Republic of China*: The personal information of natural persons is protected by law, whose rights and interests may not be infringed upon by any organization and individual). However, the information collected and processed during COVID-19 prevention and control is used to effectively delimit the scope of prevention and control, avoid the excessive diffusion of the virus, and provide timely treatment for infected people. In this case, personal information not only carries the personal information security and interests of epidemic-related individuals but also concerns public health and even national security [24], which has the attributes of compound legal interests: On the one hand, it is necessary to make public the personal epidemic-related information of confirmed and suspected cases, especially their whereabouts, to ensure the lives and health of people; on the other hand, personal epidemic-related information contains a large amount of sensitive information and personal privacy, whose collection and release will inevitably affect the rights and interests of people involved in the epidemic [4]. Under such circumstances, conflicts appear between personal and public interests in epidemic-related information.

The first is the conflict between public health security and personal information protection [25]. Characterized by human-to-human transmission and strong infectivity, the COVID-19 virus will have a significant impact on the whole of society if it spreads. As mentioned above, China has a high population density and a large number of susceptible groups including the elderly and children, in which the COVID-19 virus becomes even more threatening. Confronted with the epidemic, China has thus taken it as a common problem faced by the whole society and adopted a holistic approach to comprehensively prevent and control it in terms of prevention and control policies. Through the mastery of personal epidemic-related information, health and epidemic prevention departments can effectively describe the spread scope of epidemic-related risks, delineate prevention and control areas, and avoid the further spread of risks. At this point, personal epidemic-related information involves multiple stakeholders, such as individuals, the state, and the public. Personal interests are not the only object protected by legislation. Therefore, the limitation of some personal information protection interests is justified [26]. The exercise of individuals’ right to know, decide, and refuse, which is stipulated in the *Personal Information Protection Law* to protect personal information, needs to be restricted (Article 44 of *Personal Information Protection Law of the People’s Republic of China*: Individuals shall have the right to know and decide the processing of their personal information, restrict or refuse the processing of their personal information by others unless it is otherwise prescribed by any law or administrative regulation). Information collection subjects may collect and release relevant personal epidemic information without consent even though it may involve sensitive information or personal privacy.

The second conflict is between the right of the public to know and the protection of personal information. In view of the infectivity of the COVID-19 virus, the general public has legitimate reasons to hope to obtain as much information as possible related to the progress of epidemic prevention and control in the whole society and community, including the current situation of the epidemic, the degree of control, the relevant information of confirmed, asymptomatic, and suspected cases. as well as other groups, particularly illness state (personal medical information), travel information, the scope of epidemic-related risks, etc., to arrange their studies, work, life, and other matters, and effectively avoid the potential risk of infection. Additionally, fully ensuring the right of the public to know is conducive to dispelling rumors. At the beginning of the COVID-19 outbreak, online rumors were rampant and even caused social panic for a time due to the insufficient understanding of the virus, the unclear situation of the epidemic, and the boosting of the Internet, we-media, and other media. Disclosing government information and data and protecting the right of the public to know is the best “specific remedy” to control online rumors [27]. For individuals, however, the information collected during epidemic prevention and control includes personal private living space and activity information, involves the personality rights and interests of individuals, and originally places a reasonable expectation that personal private life will not be disturbed and can be peaceful. In the context of epidemic prevention and control, such an expectation conflicts with the demand of the public for the right to know.

Under the influence of double-edged swords such as big data tools and artificial intelligence technology, the above conflicts imperceptibly increase the risk of personal information being infringed upon [4]. According to the announcement of the Ministry of Public Security of China, in 2020, Chinese public security organizations dealt with more than 1500 people who violated the legal rights of epidemic-related personal information of citizens by sentencing public security penalties accordingly and notified relevant departments to give more than 430 people party and government disciplinary sanctions [28]. For example, according to the report of the official website of the Political and Legal Commission of the CPC Central Committee “China Chang’an” [29], on 23 December 2020, Beijing police of Shunyi District received a call from someone alleging that his family members’ information was leaked and spread on multiple social media apps, which had an impact on their lives and work. According to the police investigation, an employee of an aviation security company named Liu took a private photo of the epidemiological investigation report of the epidemic-related person during his work and sent it to the WeChat group, which led to the disclosure of the name, ID card number, home address, work unit, mobile phone number, and other private information of the epidemic-related person and his family members and colleagues. On 24 December, Shunyi police punished Liu with administrative detention according to the law. In addition, according to the news on the Sohu website [30], on 21 February 2020, Ningbo City announced a new case of COVID-19 in Beilun District, and the personal information of the patient and his relatives was also disclosed on social media, including the names, ID number numbers, photos, and mobile phone numbers of the patient and her husband, father-in-law, and son, as well as the private information of several relatives. According to the investigation, a local auxiliary police officer and a village cadre obtained this information in their work and spread it through WeChat. After that, the local police imposed a penalty of administrative detention for six days and a fine of RMB 500 yuan. Among them, the village cadres received a serious warning from the CPC, and the auxiliary police were relieved of the employment relationship by the employer.

## 3. Optimization Paths of Personal Information Processing in COVID-19 Prevention and Control in China

Over the course of the three-year epidemic prevention and control, people’s understanding of the virus, its transmission characteristics, infection channels, and other aspects gradually became deeper and clearer, which constantly clarified how to control infection sources, cut off transmission chains, identify the scope of potential risks, and take treatment measures in this process. During the three years of fighting the COVID-19 virus, the collection and processing of personal information in China gradually transitioned from the initial disorder and chaos to the current orderly, legal, and effective situation, continuously optimizing the processing paths of personal information.

### 3.1. Problems in the Early Information Collection and Transmission of COVID-19 Prevention and Control

In the early days of the epidemic, China was in the process of trial and error regarding how to collect personal information, determine the scope of the collection and the subjects with the right to collect information, deal with the collected information, and other issues from the perspective of handling public health events in the face of the COVID-19 virus with rapid transmission speed and not fully controllable consequences. In this process, certain problems such as the imperfect protection of personal information subjects appeared in the early phase of the “joint prevention and control” mechanism of the Chinese State Council [31].

First, too much personal information collected and multiple collection channels give rise to the unnecessary over-exposure of personal information [32]. At the early stages of the epidemic, it was necessary to collect a mass of personal information based on the requirements of “joint prevention and control” and from the perspective of “preventing input, output and diffusion”, but the requirements for the subjects whose information should be collected and the scope of the collection were unclear. As a result, a variety of collection methods emerged in the short term—the co-existence of the writing method, the electronic method through software, showing identity cards and recording information, etc. Personal information such as name, age, registered residence and residence address, health information such as past medical history and whether to seek medical advice, or relevant information including the history of travel and residence in affected areas and recent travel history were all collected [33]. Furthermore, a good deal of unnecessary information was collected. As the article on the official website of the Office of the Central Committee of the Communist Party of China’s Cyber Security and Informatization Commission criticizes: How Can We Collect Citizen Information in the Name of Epidemic Prevention? [34]. The article points out that for a period of time, citizens’ personal information was collected and used at will. For many people, every corner of their lives, e.g., the time they get up, their commute track, search records, consumption preferences, restaurants they often go to, strolling routes, and receiving addresses were observed, recorded, and analyzed by thousands of eyes, causing great hidden dangers. In addition, there is also a commentary on Sina.com asking what is the purpose of providing political appearance and education background in the personal information collected in the name of epidemic prevention? [35]. Such problems caused by the collection of too much information and the large scope increased the risk of personal information disclosure. Some people in seriously affected areas were discriminated against, prejudiced, and even condemned owing to the disclosure of such information. The privacy of citizens was violated.

Second, the diversified subjects of information collection and processing led to a too broad scope and made it difficult to distinguish truly authorized subjects from unauthorized ones, causing the risk of personal information protection. At the early stage of epidemic prevention and control, personal information was collected and processed by a variety of parties to achieve quick screening and identify high-risk groups, isolated cases, close contacts, etc. Information was collected in different ways and at different densities by disease prevention and control institutions, public security organs, government departments at all levels, grassroots self-governing organizations such as neighborhood and village committees, community property organizations, public place operators, various employers, educational and medical institutions, etc. At that time, it was hard for individuals to distinguish which subjects were authorized. As a consequence, personal information was illegally collected under the pretext of the epidemic, which exerted an influence on the security of personal information. In a case heard by the court of Lianshui County in Jiangsu Province of China, the defendant was accused of using the website “www.mikecrm.com” to create a link named “Lianshui County Protective Mask Reservation Service” on 7 February 2020, and releasing it through his social media app. By 1:00 on 9 February, the defendant had illegally obtained more than 4730 pieces of citizens’ personal information [36].

Third, electronic data and information technology not only provide convenience for information collection but also channels for information leakage. The diversity of information collection and processing subjects and the dispersion of collection channels lead to the opening and rule failure of information processing chains and the decentralized transmission of massive non-desensitized information. Thanks to the development of communication tools and the widespread use of social media software, a certain piece of information will form a situation of decentralized widespread transmission and leakage once sent through social media software. In the early days of the epidemic, some epidemic information was spread extensively and disorderly as people were highly concerned with and sensitive to epidemic-related information. According to the information circular issued by Guilin Public Security Bureau of Guangxi Province on 8 December 2021, on 7 December 2021, due to the need for epidemic prevention inspection, the wrongdoer named Wei received an epidemiological investigation form sent by his superior, but he unlawfully forwarded the form to the chat group of his social media app to his colleagues unrelated to his work. Afterward, another person in the colleague group forwarded the screenshot of the epidemiological investigation form to his classmates, causing the contents involving sensitive information such as the identities of people involved in the form to be continuously forwarded and spread, which produced adverse social impacts [37]. Thus, unlawful information spread leads to the risk of disclosing information about subjects’ privacy and spreads various kinds of malicious rumors. In a civil tort liability dispute case heard by a district court in Chongqing in China, the defendant was accused of obtaining the Customer List of South American White Shrimp Purchased by Chongqing through illegal means without the consent of the plaintiff and the authorization of the competent department, and publishing it on its social media official account without authorization and providing it to the public for free download. The above list contained the detailed and true personal information of more than 10,000 people including the plaintiff. The article released by the defendant company spread rapidly on the social media platform in the neighborhood where the plaintiff lived, causing great panic [38].

Fourth, the final method of processing information was unclear after the collection of vast quantities of information, increasing the ongoing risk of personal information protection. In the process of COVID-19 prevention and control, people were asked to provide personal information about residence, travel, medical care, schooling, work, and other aspects. This type of information is also used in various situations, including the query of activity trajectories, spatiotemporal intersection, medical information, vaccination status, information about co-residence, etc. The parties conducting the collection and the collecting channels of such information are also different. However, information providers have no control over the processing methods and results of the collected information, thus giving rise to the following questions: Will relevant information be retained, destroyed, or used for other purposes after being obtained? How do information subjects control it if the information is used for purposes other than epidemic prevention? Should attention be paid to the timeliness of information if it is used for epidemic prevention? An example in practice shows that the previous personal information of a person was used for epidemic prevention after a long time and thus caused unnecessary difficulties for her [39], reflecting problems such as the lack of standardized operation for the processing procedures of collected information and delayed information updating and information abuse.

Fifth, the remedies for the infringement of personal information rights and interests are not well implemented. In the early stage of epidemic prevention and control, the main legal basis for relevant prevention and control measures was existing legislation on public health emergencies, including the *Emergency Response Law*, *Emergency Regulations on Public Health Emergencies Law of the People’s Republic of China on Prevention and Control of Infectious Diseases*, etc. Provisions thereof were mainly formulated against administrative authorities and various medical institutions. For violations of the prevention and control policies, they are mostly dealt with through ex post relief. For instance, the administrative organs deal with the violator by means of administrative penalty. Nevertheless, the scope of punishment was limited. Only the primary violators were punished, but no appropriate punitive measures would be taken for individuals or groups participating in the illegal information transmission. The people whose personal information was poorly treated were still infringed upon by illegal transmission.

Certainly, the protection of personal information is not a concern only against the backdrop of epidemic prevention. “Being digital” is an important feature of the times in the present information society [19]. This paper emphasized that the personal information obtained in the context of early COVID-19 prevention and control was sensitive, systematic, and easily identifiable, which caused the risk of fully disclosing the personal information of people involved in the epidemic, and easily damaged their reputations and physical and mental health or led to their discriminatory treatment, potentially endangering their personal and property safety. As the old Chinese Saying says, lessons learned from the past can guide one in the future. Although with the end of dynamic zero-COVID-19, China’s epidemic prevention and control no longer place the collection of epidemic-related personal information in an important position, it is still necessary for us to rationally reflect on the epidemic prevention and control process in the past three years, and to attach importance to how to better collect and deal with personal information in public health emergencies and other similar emergencies involving a wide range of groups, so as to systematically improve the protection of personal information in China.

### 3.2. Reasons for the above Problems

Based on the above analysis, the protection of personal information during the “dynamic zero-COVID-19” epidemic prevention and control contained the following main pain points: Firstly, the basic principles of personal information protection were violated, such as the principles of anonymity, purpose limitation, and balance, due to the incompleteness of the awareness and means of protection in the process of personal information processing. Secondly, the degree, subjects, and methods of responsibility were ambiguous, leading to the failure to take timely remedial measures and provide victims with appropriate ways to safeguard their rights after the leakage of personal information. Thirdly, the practice of adopting “one-size-fits-all” administrative punishment to pacify people could produce good social effects in the short run, which, however, not only put great pressure on public opinion and work on relevant departments but also was not conducive to realizing the virtuous circle of personal information protection in the long run.

The reasons for the above problems include the imperfection of the legal system and the influence of traditional Chinese social concepts. Concerning its legal system, China has already formulated laws and regulations such as the *Emergency Response Law*, *Emergency Regulations on Public Health Emergencies Law of the People’s Republic of China on Prevention and Control of Infectious Diseases*. The legal system to deal with public health emergencies has taken shape and formed the framework of divisions that are oriented by local people’s governments, according to which health administrative departments authorize disease prevention and control institutions and various medical institutions to collect and release epidemic-related monitoring information. These relevant legislations make all epidemic prevention and control policies and measures legally rational and legitimate. However, the large-scale and comprehensive COVID-19 outbreak under the “joint prevention and control” system has its own structural risk, an inescapable risk in a “preset environment” [40]. In response to the epidemic, China adopted the system of joint and mass prevention and control and the method of “dragnet screening” corresponding to “national participation” and “precise prevention and control” [17], which lengthened the chain of information collection and processing, and highlighted the weaknesses and deficiencies of relevant laws and regulations mentioned above in the procedures for the publication and release of epidemic information, the scope, method, channel, time limit of information release, and other important issues, and the lack of detailed provisions on how to exercise and delegate power.

In the process of COVID-19 prevention and control, the scope of information collection subjects was large, and the chain of information collection was long. Not all the links involved were administrative subjects. Furthermore, the scope of subjects stipulated by the aforementioned relevant laws and regulations in response to public health events was not fully covered. For example, grassroots community workers would collect and sort the basic information, relevant travel information, and activity trajectories of community residents based on the needs of prevention and control work, in addition to community health service personnel and medical unit staff gaining access to and obtaining the epidemic-related medical information of residents. Enterprises and public and educational institutions would collect and sort the basic, travel, health, and other information of their staff, students, or cohabitants. Some managers of public places even needed to collect basic, medical, and other information about people moving in and out of these places. The chain of information collection and processing was long and not closed-ended. Meanwhile, not all corresponding subjects were professional or administrative staff, and operation specifications and requirements for corresponding information collection and sorting were lacking, contributing to too many uncontrollable factors in all links of personal epidemic-related information collection and processing and making it difficult to eliminate the risk of personal information being wrongly dealt with.

From the angle of the traditional concept, on the other hand, traditional Chinese society is a society that is not only too familiar to allow privacy but also deeply influenced by Confucian culture. Mr. Fei said, “Law will not happen in a rural society … where people get familiar with and then trust each other” [41]. The analysis of the problems in China cannot ignore the influence of this traditional concept on the behavioral pattern of people. By comparison, the concept of privacy in western countries is young in China in which individual privacy rights and related privacy interests are diluted and taken for granted in reality under the influence of the traditional ethical thought of safeguarding national and social interests [42]. In a nepotist society, moral concepts have a profound influence on social governance, and the consciousness of paying attention to the interests of the whole is ingrained in the minds of Chinese people. People’s concept of privacy is downplayed under the influence of this value tendency and historical and cultural environment. On account of the lack of accumulation of historical habits and sufficient public discussions, both personal information and privacy rights lose when measured against public interests and lag behind when compared with other private rights [19]. Hence, the protection of personal information itself in China has a long way to go.

### 3.3. Optimization Evolution of Personal Information Processing in COVID-19 Prevention and Control in China

In the early phase of the fight against the COVID-19 virus in China, due to insufficient preparation for the unexpected outbreak of the virus, in order to control the spread of the virus as efficiently as possible, provide infected people with timely treatment, and reduce the damage and impact of the virus on the whole society, early epidemic prevention and control attempted to obtain and process all epidemic-related information by establishing perfect information networks, which overlooked the risks in the whole processing chain to some extent and resulted in the aforementioned phenomena of infringing upon personal information rights and interests. However, people started to reflect on how to better protect personal information rights and interests in the process of dealing with the virus and tried to avoid the structural risks arising from the institutional design and operational process of prevention and control linked to the proceeding of epidemic prevention and control.

The path taken by China to optimize the protection of personal information can be explored from the two perspectives of legislation and specific operation.

Firstly, in terms of legislation, on 4 February 2020, the Office of the Central Cyberspace Affairs Commission issued the Notice on Personal Information Protection and the Use of Big Data to Support Joint Prevention and Control, requiring that the collection and use of personal information in epidemic prevention and control should comply with relevant laws and regulations and national standards and clarifying the principle of adhering to the minimum scope, etc. [4], thereby specifically stipulating the requirements for improving personal information protection in epidemic prevention and control in the form of departmental regulations from the perspective of administrative authorities. Additionally, The Civil Code of the People’s Republic of China, which was formally promulgated and implemented on 1 January 2021, provides normative requirements on information protection for three subjects, which are personal information processors, state organizations, administrative organizations, and medical institutions, and violators of such norms will bear civil tort liability. In view of this, compared with the previously existing public health emergency disposal of the relevant administrative laws and regulations, from the perspective of civil law basic provisions, The Civil Code of the People’s Republic of China stipulates the requirements for the protection of personal information rights and interests and directly gives private legal means to information subjects to protect their information rights and interests, aiming to facilitate the information subjects to legally protect their legitimate information rights and interests. Thirdly, another important legislative achievement is the Personal Information Protection Law of the People’s Republic of China promulgated and implemented on 1 November 2021. Based on the basic provisions of personal information protection in The Civil Code of the People’s Republic of China, this Law further details the provisions of personal information protection. It outlines detailed regulations on the excessive collection of personal information, collection and acquisition methods of sensitive personal information, and information subjects’ right to know information processing activities and their rights to collected information, integrates the provisions on the rights, obligations, and responsibilities of relevant parties, and stipulates clear and specific obligations and responsibilities for personal information processors, managers of public places, subjects providing important Internet platform services, and departments performing personal information protection duties.

All of the above legislations perfect the legal system of personal information protection and help to improve the insufficient protection of personal information in the current epidemic prevention and control process, especially the establishment of private law relief approaches, thereby enabling the information subjects to obtain means of safeguarding rights and being more conducive to realizing the protection of interests in terms of individuals.

Secondly, from the perspective of specific measures for the collection and processing of personal information, there is improvement in both the collection process of information and the release channel of information.

In the early stage of epidemic prevention and control, there were many problems in personal information collection, such as excessive collection contents, multiple collection subjects, and collection channels [43]. By presenting personal identification documents and registering detailed personal information at home, much information not necessary for epidemic prevention and control is overexposed and may be exposed to the risk of illegal disclosure due to the unclosed information processing chain [10]. With the furthering of epidemic prevention and control, in terms of the specific operation mode of personal information collection, various regions in China have adopted a unified electronic information collection method—to collect and store the basic information related to personal epidemic information in the form of a two-dimensional code (QR code) through the national unified or provincial unified electronic health passcode, travel card, place code, and other programs. These QR codes can be scanned to obtain the relevant information in public places, for the purpose of nucleic acid test registration, or travel tracking. During this process, the operators of intermediate links such as those scanning and collecting information are unable to obtain personal information stored in the QR code, and all personal information is stored in the unified and closed-loop system. This approach can largely reduce the personal information leakage risks caused by unnecessary collection contents and multivariate main collection bodies and collection channels. Moreover, the principle of “least harm” in the handling of personal information can be realized.

In terms of the use of the QR code, many details also facilitate the judgment of individual epidemic risk in daily prevention and control. For example, the health code can indicate whether the holder is an infected person or has epidemic risk by changing the colors to green, yellow, and red. In the process of daily epidemic prevention and control, managers of public places can easily understand the epidemic risk of people entering and leaving the place through the colors and control the epidemic risk of the whole public place. The electronic processing mode of place code simplifies the registration procedure of personnel access and facilitates the epidemiological investigation procedure in the presence of epidemic risk. Electronic data can accurately record the detailed whereabouts of a person so that the follow-up epidemic prevention and control work can be carried out rapidly and the efficiency of epidemic prevention and control can be improved. 

Furthermore, for low-risk areas, on some daily occasions where there is no need to display personal details but rather only a need to confirm whether an individual is at risk of being involved in the epidemic, a variety of grass-roots units have simplified the confirmation procedure and can achieve normal passage by only showing some simple proof. Among them, the most typical example is the application of nucleic acid test “paste”. In the dynamic zero-down epidemic prevention and control state, most grassroots communities in China regularly conduct nucleic acid tests for all their staff or key groups in order to screen out the risk of COVID-19. As a normal epidemic prevention measure, people need to show their health code to register their identifying information and complete the nucleic acid test, and therefore they can quickly locate the source of nucleic acid samples and complete the rapid flow adjustment work in the case of abnormal test results. However, in low-risk areas, the grassroots community adopts the method of issuing nucleic acid detection “paste” to prove their completed conventional nucleic acid detection in order to facilitate people’s daily travel. In some daily life situations, such as the community residence, supermarket, etc., people can normally travel by only showing the “paste” instead of showing other substantial identity information, thereby facilitating people’s daily life and travel. This is not only for the protection of personal information rights and interests but also for the necessity and legitimacy principle of realizing personal information collection and processing.

Thirdly, with the release of epidemic-related information, the protection of personal information has also been optimized with the implementation of epidemic prevention and control in China. In the early days of COVID-19 prevention and control in China, people were highly sensitive to people and information related to COVID-19 and were eager to learn information concerning local or neighboring regions, therefore finally determining their own risks. Driven by this universal social psychology, much information related to the epidemic was disclosed or spread without authorization in the early chain of information collection and processing [4]. Some of the information spread involved personal privacy information not related to the epidemic, some involved less accurate information that had no final verification, and some was even related to false information and rumors [44]. These factors not only violate the relevant personal information rights of the individual but also violate their privacy and can even cause panic due to the spread of rumors. After the Notice on Personal Information Protection and the Use of Big Data to Support Joint Prevention and Control was released on 4 February 2020, the relevant administrative departments began to focus on strengthening the collection, processing, and unified release of epidemic-related information. For the release process, the National Health Commission of the People’s Republic of China and the local health commissions conducted a unified arrangement for epidemic information collection and processing. From 22 January 2020, the data of confirmed cases and suspected cases in all provinces of the country were uniformly released by the National Health Commission every day [45]. With the optimization and adjustment of the overall epidemic prevention and control policy in China, on 25 December 2022, the National Health Committee of the People’s Republic of China made a statement that it would no longer release the daily epidemic information. Thus, it ended its mission of uniformly releasing authoritative official data on the epidemic situation in the past two years and eleven months [46]. In addition, all the published information was desensitized and presented anonymously, and thereby people could obtain the latest, most accurate, and authoritative data through its website, without being disturbed by the chaotic information spread privately with nowhere to be verified. Just as “sunlight is the best preservative”, this official information release method effectively promoted the legal implementation of COVID-19 prevention and control.

## 4. Reflection and Forward-Looking on Personal Information Protection during Public Health Emergencies in China

In the early period of COVID-19 prevention and control in China, due to insufficient preparation for the outbreak of the public health emergency, the entire Chinese society experienced a period of confusion and disorder in the processing of epidemic-related information. As mentioned earlier, the concept of “focusing on information collection and ignoring information management” behind the concept of “all for epidemic prevention and epidemic relief” led to a large number of excessive collection contents and multiple collection subjects and collection channels of personal information. Subsequently, the electronic information collection method was implemented nationwide, optimizing the information collection process and release channels. The application of electronic data technology, represented by QR codes, has become an innovation in China’s response to public health emergencies. With the help of data technology, real-time epidemic monitoring, key screening, and effective prevention have been realized. However, the optimization of this information processing technology is continuous, and the application of new means also brings some new problems. At present, the focus of COVID-19 prevention and control in China has shifted from “prevention” to “protection” and “treatment”. The collection and processing of personal information are no longer focused on prevention and control. However, the handling of similar public health emergencies now and in the future can expect the support of big data and information technology. Therefore, this is a good time for serious summarization and reflection, and we need to address the multiple risks in the public health security and personal information protection fields, as well as the shortcomings in protecting personal information security in practice. Therefore, we should summarize and reflect on the measures we have taken in the face of the new situation and the experience and lessons we have experienced in a timely manner and provide useful suggestions for the Chinese government to strengthen personal information protection in the future data society governance, so as to help improve the data society governance capability.

### 4.1. Strengthen the Proportionality between Personal Information Protection and Restrictions Imposed by Public Power—Exercise of Public Power Should Be Restrained

Due to the urgent reality at the early stage of COVID-19 prevention and control, when electronic information collection means, such as QR codes, were introduced, their justification has been in a state of vacancy, which needs to be supplemented in the present stage. The essence of QR code usage is the restriction of public power on private rights. On the one hand, the government’s promotion of QR codes aims to give priority to public health security, aiming to protect the most basic right to life of individuals, thus making it legitimate to restrict private rights. However, on the other hand, the legitimacy of public power intervention must also be subject to proportionality review. Its restrictions on personal information rights and interests must comply with the principle of proportionality. The personal information security risks caused by health codes should be controlled within a reasonable range [47].

QR codes are a data innovation tool generated in the specific context of COVID-19 prevention and control, and the personal information involved in it should be used in the specific field of epidemic prevention. Due to the huge potential of personal information resources in social management and government services coupled with the absence of supervision, many governments tried to break through the purpose of epidemic prevention and made use of QR codes outside the field of epidemic prevention. Among them, the “Colored Code” of Hangzhou City [48] and the “Civilization Code” of Suzhou City [49] are the most criticized. The above practices tried to break through the scene limitation in epidemic prevention and control and use personal information for personal health rating and civilization scoring that had nothing to do with epidemic prevention and control. It is not only difficult to achieve the preset purpose, but it would trample on personality equality and personal freedom, and the resulting blocked implementation of both was expected. The usage of personal information for other purposes apart from the specific context of epidemic prevention not only lacks a legal basis but would also impose excessive restrictions on the legal interests of personal information protected by the Civil Code of China.

The exercise of public power should respect and protect privacy rights. As for the institutions that undertake administrative functions, such as state organizations, their collection and processing of personal information is different from other organizations such as natural persons, enterprises, social organizations, etc. The collection and processing thereof are based on their legal responsibilities and the needs of public interests. Their behavior is mandatory. Once this public power is abused, it would pose a greater threat or harm to the information subject. The collection methods of electronic information such as QR codes generated in public health emergencies involve the most sensitive personal information. For the purpose of giving priority to the protection of public health and safety, such electronic information collection methods are essentially mandatory, but they only solve the legitimacy problem of restricting the rights and interests of personal information, which must be exercised under the principle of proportionality, requiring the public power to exercise a high degree of restraint, and should not be used too generically.

### 4.2. Strengthen the Protection of Personal Information Rights and Interests—Private Rights and Interests Need to Be Attached Importance

Personal information protection first involves personal interests, and safeguarding personal interests is the logical starting point of personal information protection [50]. From the specific provisions of the Civil Code and the Personal Information Protection Law of China, the core idea of the protection of personal information rights and interests is to strengthen the control of the personal information subject over its information. In principle, personal information processing must be open and transparent and approved by the personal information subject. However, due to the urgency of epidemic prevention, the operation of QR codes escaped the review of legality. The principle of informed consent for personal information protection was almost non-existent, and many personal information processing activities were dissociated from legal constraints.

As mentioned earlier, QR codes can indicate the epidemic-related risk of its owner through the change in color, but in practice, the judgement standard and algorithm rules for coloring the QR codes are not transparent, and the coding rules especially are completely in the dark box. In June 2022, the incident of “depositors of banks in Henan Province were given red QR codes” aroused widespread concern in society [51]. The violators illegally “gave red codes” to the depositors’ “QR codes” for improper purposes, indirectly restricting their freedom. This kind of indiscriminate abuse of personal information transferred by people to cooperate with national epidemic prevention is an infringement of peoples’ personal information, which also reflects the fact that people have no chance to agree to or even know of the follow-up processing methods of the collected information, but can only passively bear the consequences of information abuse and infringement on their rights and interests because the principle of informed consent for personal information protection is ignored. In fact, in the context of epidemic prevention, for the sake of public health and safety, the use of QR codes is essentially mandatory, so the processing of personal information is exempt from the consent rule here [47], but the informed rule plays an indispensable role in the defense and supervision of personal information protection [52]. When dealing with personal information obtained based on electronic information collection, relevant personnel shall fully notify and explain the category, necessity, purpose, and information management methods (including coding algorithm logic and data protection technology) of personal information processing, so as to provide behavior guidance for the public.

### 4.3. Careful Treatment of the Balance between Public Interests and Personal Interests Protection—Avoid Excessive Infringement of Personal Interests in the Name of Public Interests

From the perspective of traditional culture, Chinese society has long been affected by the concept of public interest first, and personal interests are often suppressed. However, with the progress of society and the improvement of China’s personal information protection legal system, public interests should not be generalized, and people’s private rights should also be reasonably protected. It is true that peoples’ private rights will be limited because of the need to prevent and control COVID-19, but the limitation should have the bottom line of the rule of law. It should be stopped to cover up excessive violations of personal legitimate interests under the “umbrella” of public interests. Careful adjustment of the boundary and tension between public power and private rights is expected, and people’s legitimate rights and interests of personal information should be protected.

In the context of epidemic prevention, the priority of public health and safety lies in the right to life. The right to life indicated by public health security is the most basic and important right of individuals, which should be given priority for protection. Since the outbreak of the COVID-19 epidemic, China has spared no effort to emphasize the prevention and control of the virus and the dynamic-zero COVID-19 policy. The starting point is also to try to control the spread of COVID-19 as much as possible under the circumstances of having no sufficient control over the infectivity, concealment, and harmfulness of the virus, to treat the infected cases in a timely manner, and to achieve all aspects of prevention, treatment, and control preparations in the time window that has been won in order to gain greater confidence in defeating COVID-19 to build a solid barrier for everyone’s right to life and health.

The government’s public power originates from and serves the private rights of its people, but the two are often in a state of trade-off. The expansion of public power usually restricts the private rights of people. In response to emergencies, despite the expansion of the government’s power and the contraction of peoples’ individual rights, the “bottom line” should still be defined; otherwise, the government’s personal data processing and information disclosure based on legitimate purposes may degenerate into excessive intervention in individual rights [53]. The simplified ranking of interests cannot provide the final answer to the conflict. We should find a balance between personal interests and public interests and promote the realization of public interests on the premise of ensuring that personal interests are not infringed upon to the maximum extent. For example, identifiable personal information shall not be disclosed in epidemic-related information disclosure. The Law on the Prevention and Treatment of Infectious Diseases of China clearly stipulates that “no information involving the privacy of the diagnosed person shall be disclosed on purpose”. Therefore, even for the purpose of better preventing and controlling the epidemic situation and protecting the public’s right to know, the government should also actively assume the responsibility to protect peoples’ basic rights (such as the right to privacy) and freedom, and should not disclose information that can identify specific individuals. This issue is not a simple interest ranking issue. Different choices need to be made for the conflicts of interests of different subjects, that is, to always compare and weigh, in this specific context, which interests are more important.

## 5. Conclusions

When public interests and the right to life and health conflict with other rights, it is legitimate and necessary to reduce and limit other rights to some extent to safeguard public interests and the right to life and health, which is a basic right [4]. However, the compromise of personal information protection for public interest indicates that relevant personal information can be collected and published within the scope that it is necessary to protect the public interest, whereas it does not mean that disorderly, excessive, and unrestricted collection, use, or even abuse of personal information can be carried out. Understanding can deepen with practice. China has largely reversed the problem of inadequate personal information protection in the early stages of epidemic prevention and control and explored the optimal path from both legislative and practical perspectives. Based on this realistic perspective, the deficiencies, causes, and methods of optimization in the future concerning personal information protection in public health emergencies are explored to provide some references for promoting the legal construction of personal information protection in the long term.

## Data Availability

Not applicable.
